# The psychosocial impacts of the 15 March terrorist attack on the Christchurch Muslim community: A descriptive, cross-sectional assessment

**DOI:** 10.1177/00048674241276802

**Published:** 2024-09-08

**Authors:** Ruqayya Sulaiman-Hill, Philip J Schluter, Sandila Tanveer, Joseph M Boden, Richard Porter, Ben Beaglehole, Shaystah Dean, Zimna Thaufeeg, Caroline Bell

**Affiliations:** 1 Department of Psychological Medicine, University of Otago, Christchurch, Christchurch, New Zealand; 2 Faculty of Health (Te Kaupeka Oranga), University of Canterbury (Te Whare Wānanga o Waitaha), Christchurch, New Zealand; 3 School of Clinical Medicine, Primary Care Clinical Unit, The University of Queensland, Brisbane, QLD, Australia

**Keywords:** Trauma exposure, psychological outcomes, Muslim population, terrorist attack

## Abstract

**Objective:**

On 15 March 2019, a white supremacist terrorist carried out sequential attacks on two mosques in Christchurch, New Zealand during Friday prayers. This resulted in the loss of 51 lives, 40 others sustained gunshot injuries, and there were approximately 250 survivors. This study aimed to evaluate the impacts on community members, assess clinical needs, facilitate access to appropriate interventions and provide insights into working with a traumatised and diverse population.

**Methods:**

This cross-sectional study used semi-structured clinical interviews and self-report measures to assess social and demographic factors, mental health disorders and well-being for adult Muslims 11–32 months post-attack.

**Results:**

A total of 189 participants completed assessments. The sample was diverse, representing 34 different ethnicities and participant proximity to the attack was complex, with personal and familial exposures. Elevated levels of psychological distress and psychopathology were found with 38% of participants reporting moderate/severe psychological distress on the Kessler-10, 39% reporting post-traumatic stress disorder on the post-traumatic stress disorder checklist-5, and 40% reporting poor well-being or possible depression on the World Health Organization-5 Well Being Index. Secondary stressors were also documented, as well as high scores for post-traumatic growth and the importance of faith.

**Conclusion:**

This study provides valuable insights into the repercussions of the Christchurch mosque attack on the affected community, describing the complexity of exposure and the substantial burden of morbidity experienced. It also highlights the high levels of social connectedness and the role of faith in promoting positive outcomes in the recovery process for this population.

## Introduction

In recent years, Western democracies have seen an increase in white supremacist ideologies and right-wing extremism, with severe consequences at times (Clark, 2020; Leander et al., 2020). One such incident occurred on 15 March 2019 in Christchurch, New Zealand, when a gunman with extreme far-right, ethno-nationalist and Islamophobic beliefs sequentially attacked two mosques during Friday prayers. This act of terrorism resulted in the loss of 51 lives,^1^ ranging in age from three to 77 years, 40 others were wounded (Auger, 2020; Royal Commission of Inquiry (RCOI), 2020; Sulaiman-Hill et al., 2021), and approximately 250 survivors witnessed the incident. It has left a lasting impact on the affected individuals and their communities (RCOI, 2020; Sulaiman-Hill et al., 2021). In an international first, the terrorist simultaneously streamed 17 minutes of the attack on social media (Macklin, 2019), resulting in additional widespread and repeated exposure. This is one of the worst mass shootings in peacetime and is unprecedented in modern New Zealand (Wilson and Thomson, 2019). Although the incident occurred at two different locations, this paper will refer to it as a single ‘attack’ for consistency.

In response, a Royal Commission Of Iinquiry (RCOI)^2^ was conducted, allowing survivors, witnesses and community members to share their experiences and describe the consequences of the attack. The accounts revealed serious ongoing physical challenges and psychological distress (RCOI, 2020). The impact on the general New Zealand population has also been far-reaching, with a notable increase in terrorism anxiety and an enhanced sense of community and solidarity reported initially, although measured psychological distress and well-being appear to have remained unchanged over time (Byrne et al., 2022).

The Muslim population in Christchurch, which comprises about 1% of the region’s population (Statistics New Zealand, 2020), is ethnically and demographically diverse. Many people have recent immigration histories (Strategic Social Policy Group, 2008) and include those from refugee backgrounds who have often been exposed to prior traumas. Given previous research and recognising the potential vulnerability of the survivors, families and community members (Glad et al., 2021; Paz García-Vera et al., 2016), we hypothesised that there were likely to be serious mental health impacts following this attack. In response, a collaborative partnership was established between local universities, Muslim community representatives and the regional health board to conduct an inclusive, trauma-informed and culturally sensitive research study (Sulaiman-Hill et al., 2021). The study aimed to assess the long-term impacts of the March 15 attack on the Christchurch Muslim community, to identify clinical needs, provide access to appropriate interventions and gain insights into effectively working with a traumatised, ethnically diverse population (Sulaiman-Hill et al., 2021). This paper describes the first phase of a planned longitudinal study, providing a comprehensive profile of the participants impacted by the terrorist attack and their measured psychological responses.

## Methods

### Study design

The study is cross-sectional and is intended to form phase I of a longitudinal study. It employed a mixed-methods design incorporating culturally acceptable self-report measures and clinician-administered diagnostic interviews. The development and design of the study, including the recruitment procedure and instruments used, are detailed in the study protocol (Sulaiman-Hill et al., 2021).

### Community involvement

To ensure cultural appropriateness and improve community engagement, research assistants (RAs), and several research team members were drawn from the Christchurch Muslim community. They were all university graduates who had a personal and professional investment in the study’s success and its impact on their community. In addition, an independent Muslim reference group, consisting of representatives from different exposure groups, ethnicities, religious leaders and Muslim health professionals, was established. Their role was to review and discuss the study format, measures and instruments and to ensure that data collection and the dissemination of findings were conducted in a culturally sensitive and respectful manner.

### Participants

Eligible participants were adult Muslims (aged ⩾ 18 years) who were present in Christchurch at the time of the terrorist attack and were residents in Christchurch at the time of the interview.

### Recruitment and interview procedure

Multiple strategies were employed for participant recruitment. Chain referrals from RAs and endorsements from the Muslim community were actively encouraged. Recruitment materials, such as flyers and posters in various languages, were distributed in community spaces and online platforms, with dedicated emails, phone lines and a website providing additional information and access points (see: https://www.otago.ac.nz/march/index.html).

Muslim RAs coordinated the self-report component of interviews and provided language support if needed. Mental health clinicians on the team conducted the diagnostic component of the interview. Assessments were conducted in person or via Zoom using questionnaires on Qualtrics (Qualtrics, Provo, Utah, USA). Participants were given the choice of completing the assessment in English or their preferred language from Arabic, Bangla, Farsi, Turkish, Somali and Urdu. Interviews began in February 2020 and continued until December 2021, spanning a period of 11–32 months after the attack.

### Self-report measures

The self-report measures were chosen for their validated effectiveness in assessing the variables of interest, including previous use with ethnically diverse groups. Several of these tools have been previously used in trauma research, ensuring compatibility with other studies. The measures assessed current social and demographic factors, including well-being, distress and coping strategies. Demographic measures included exposure to the attack, reasons for coming to New Zealand, years lived in New Zealand, proficiency in English, highest level of education and measures of previous exposure to trauma. Standardised measures examined subjective quality of life (Personal Well-being Index, PWI) (Lau et al., 2005), social support (Social Network Index, SNI) (Berkman and Syme, 1979), perceived discrimination (Perceived Discrimination Scale, PDS) (Williams et al., 1997), work and social adjustment (Work and Social Adjustment Scale, WSAS) (Mundt et al., 2002), psychological distress (Kessler-10, K-10) (Kessler et al., 2003), post-traumatic stress disorder (PTSD Checklist 5, PCL-5) (Weathers et al., n.d.), somatic symptoms (Somatic Symptom Scale 8, SSS-8) (Gierk et al., 2014), and subjective well-being and depression screening (WHO Well-being Scale, WHO-5) (Topp et al., 2015). Post-traumatic growth was measured with the post-traumatic growth inventory (PTGI) (Tedeschi et al., 2017), and religious coping used a scale developed specifically for use with Muslim populations (Religious Coping Scale, RCS-Muslim) (Adam and Ward, 2016). See Table 1 for a full description of the measures used.

**Table 1.** Self-report measures.

Measures	Instrument	Description
Mental health and well-being		
Psychological distress	Kessler-10 (K-10) (Kessler et al., 2003)	10 questions on 5-point scale (1 none of the time–5 all of the time) relating to psychological distress in previous 4 weeks
Post-traumatic stress disorder	PTSD checklist (PCL-5) (Weathers et al., n.d.) Including one additional question relating to survivor guilt	5-point scale (0 not at all–4 extremely) relating to how bothered they have been to 20 *DSM*-5 criteria for PTSD items in the past month. An additional question was added asking about **survivor guilt**
Somatic symptoms	Somatic symptoms scale-8 (SSS-8) (Gierk et al., 2014)	5-point scale (0 not at all – 4 very much) related to how bothered participants were by 8 common somatic symptoms in the previous 7 days
Well-being and depression screening	World Health Organisation well-being scale (WHO-5) (Topp et al., 2015)	5 well-being items on 6-point scale (0 at no time–5 all of the time) to give a raw score. This is multiplied by 4 to give a final score out of 100
Quality of life, social support and discrimination		
Quality of life	Personal Well-being Index (PWI) (Lau et al., 2005)	Rating scale (0 completely dissatified-10 completely satisfied) Q1 relates to Satisfaction with life as a whole Q2-9 relate to current satisfaction with quality of life domains.
Social support	Social Network Index (SNI) (Berkman and Syme, 1979)	Part 1 Asks about group participation & frequency Part 2 scale measuring number of social contacts in different contexts
Perceived discrimination	Perceived discrimination scale (PDS) (Williams et al., 1997)	5-item scale recording experience of perceived discrimination in previous 3 months in 7 situations, and how they felt (1 not at all upset–5 very upset)
Work and social adjustment	Work & social adjustment scale (WSAS) (Mundt et al., 2002)	5 questions with 9-point scale (0 not impaired–8 very severely impaired) relating to ability to perform day to day tasks
Post-traumatic growth and religious coping		
Post-traumatic growth	Post-traumatic growth inventory (PTGI) (Tedeschi et al., 2017)	21 items on 6-point scale (0 did not experience–5 experienced change to a very great degree as a result of the crisis) Report as total score and subscales.
Religious coping	Religious coping scale – Muslim (Adam and Ward, 2016)	14 items on 5-point scale (1 not at all–5 a lot). Participants indicate whether they have engaged with behaviours or thoughts to deal with feelings of distress in previous 3 months.

### Secondary stressors and support services

Lists of potential secondary stressors and helpful supports were compiled and evaluated following community consultation. Participants were asked to indicate the services they accessed and any activities they attended, and provide feedback on their helpfulness.

### Clinical interview

A semi-structured diagnostic interview, based on the Mini-International-Neuropsychiatric-Interview (Sheehan et al., 1998), was used to assess the presence of mental health disorders prior to the attack, in the period since the attack, and at the time of the interview. This was conducted by a mental health clinician from the research team. Unless requested by the participant or required for language support, RAs were not present during this part of the interview to ensure participant confidentiality and encourage open communication. Following the interview, all cases were discussed with one of the team psychiatrists, and if required, referrals were made to appropriate organisations.

### Research ethics and consent

Ethical approval was granted by the New Zealand Health and Disability Ethics Committee (HDEC Reference: 19/NTA/147). Conduct of the study complied with the ethical standards for human experimentation as established by the Helsinki Declaration. All methods were performed in accordance with HDEC’s relevant guidelines and regulations. The study only included those people who provided written informed consent. They could choose to not participate or to withdraw at any time without penalty. Gift vouchers of $50 NZD were provided as a token of appreciation.

### Statistical analyses

Reporting of study findings was informed by the STrengthening the Reporting of OBservational studies in Epidemiology (STROBE) guidelines (Von Elm et al., 2007). All analyses were conducted using Stata SE version 17.0 (StataCorp, College Station, TX, USA). As the analysis in this paper is descriptive, summary statistics and distributions are provided. Means (standard deviations, SD) and medians (25th percentile, Q_1_; 75th percentile, Q_3_) are used for continuous variables. Given the sensitivity and potential identifiability associated with small cell sizes (less than 5% of the study population), some numerical information is withheld.

## Results

### Participant flow

**Figure 1.**
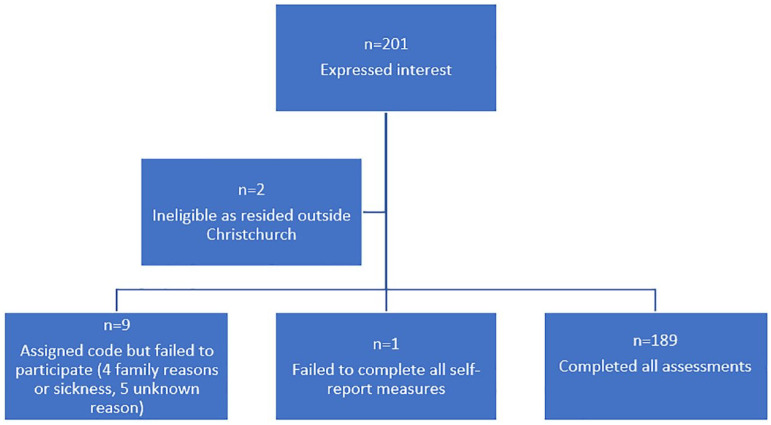
Participant flow diagram.

The lack of a sampling frame and the unavailability of a victim list necessitated the use of chain referral methods for recruitment, making it difficult to estimate the reach of the study. Of the 201 individuals who contacted the study team, 12 were ineligible or failed to complete the assessments, leaving a final analytic sample of *n* = 189 participants. The Participant Flow Diagram (Figure 1) provides a detailed breakdown of participants and non-participants throughout the study.

Interviews were conducted a median time of 2.1 years (range: 11–32 months) after the attack, with 47% completed in the first year, between March 2020 and February 2021. A 3-month hiatus in interviews occurred during this period due to the implementation of COVID-19 lockdown measures.

### Participant demographics

The sample included participants ranging in age from 19 to 74 years, with a median age of 39 years (Q_1_ = 31, Q_3_ = 48 years), and 60% identified as female. The majority (89%) were born outside New Zealand and had lived in the country for a median of 11 years (Q_1_ = 5, Q_3_ = 14 years). Many had immigrated (34%) or arrived through the refugee programme (28%). The sample was ethnically diverse, with 34 different ethnicities represented, including African, European, Asian and Middle Eastern heritage (see Table 2).

**Table 2.** Sociodemographic characteristics of sample (*n* = 189).

Characteristic	n	(%)
Gender		
Male	75	(40)
Female	114	(60)
Age (years)		
18–29	42	(20)
30–39	57	(30)
40–49	49	(26)
50–59	25	(13)
60 and above	16	(8)
Ethnic origin		
Afghanistan (includes Hazara, Pashtun, Tajik, etc.)	41	(22)
Middle East (includes Egypt, Algeria, Turkey, Iran, etc.)	38	(20)
Indian (includes those of Indian ethnicity from India, Fiji, and elsewhere)	25	(13)
African (includes sub-Saharan Africa, e.g., Somalia and South Africa)	21	(11)
Pakistan	15	(8)
Bangladesh	14	(7)
South East Asia (includes Malaysia, Indonesia, Singapore)	14	(7)
European (NZ-born and migrants) and Others (Māori, Pasifika)^a^	21	(11)
Self-reported reason for coming to NZ		
Born in NZ	20	(11)
Migrant	64	(34)
Refugee or refugee family reunification	53	(28)
Visitor	11	(6)
Other (student or spouse of student *n* = 26, work *n* = 7, various other reasons all involving very low numbers, e.g., *n* < 5)	41	(22)
Self-reported language proficiency		
* Spoken English*		
Very good	88	(47)
Good	52	(28)
Average	33	(17)
Poor	6	(3)
Very poor	10	(5)
* Written English*		
Very good	81	(43)
Good	50	(26)
Average	24	(13)
Poor	9	(5)
Very poor	7	(4)
Education		
No formal qualification (attended school, ESOL class)	24	(13)
Secondary school (e.g. NCEA, IB diploma, overseas school qualification)	27	(14)
Tertiary qualification (e.g. Certificate, Diploma or Trade qual. <3 years)	29	(15)
Bachelor degree (3- to 4-year course)	53	(28)
Post-graduate degree (PG diploma, Master, Doctorate)	56	(30)

a European includes NZ-born pakeha and migrants with European ethnic backgrounds. It has been combined with the small number of participants from the Māori & Pasifika groups to protect identity.

The majority of interviews (80%) were conducted face-to-face, and the remainders were online. Assessments were primarily conducted in English (71%), although Farsi (12%), Arabic (8%), and Bangla, Somali, Turkish, or Urdu (7%) languages were also used. English proficiency was generally high, with 92% self-assessing their spoken English as average to very good, and 82% rating their written English similarly. Educational attainment was also high, with 73% having at least a tertiary qualification, and 30% holding a post-graduate degree. Employment status varied, with 36% employed full-time, 15% employed part-time, 25% unemployed, and 8% not in the labour force. In addition, 13% were enrolled in full-time study, with 4% studying part-time.

### Previous exposure to traumatic events

Most participants (80%) reported exposure to at least one major traumatic event prior to the mosque attack, and 10% reported exposure to three or more. These included living in a war zone or being exposed to military conflict (30%) and experiencing natural disasters (60%), with 44% living through the 2010–2011 Canterbury earthquake series (Ardagh et al., 2012), and 19% exposed to other natural disasters. Childhood adversity before the age of 16 years (such as neglect, bullying, physical, or sexual assault) was reported by 13%. Physical or sexual assault at or after the age of 16 years (less than 5%), serious physical accidents (16%), and other traumas (16%) were also reported.

### Incident exposure

**Figure 2.**
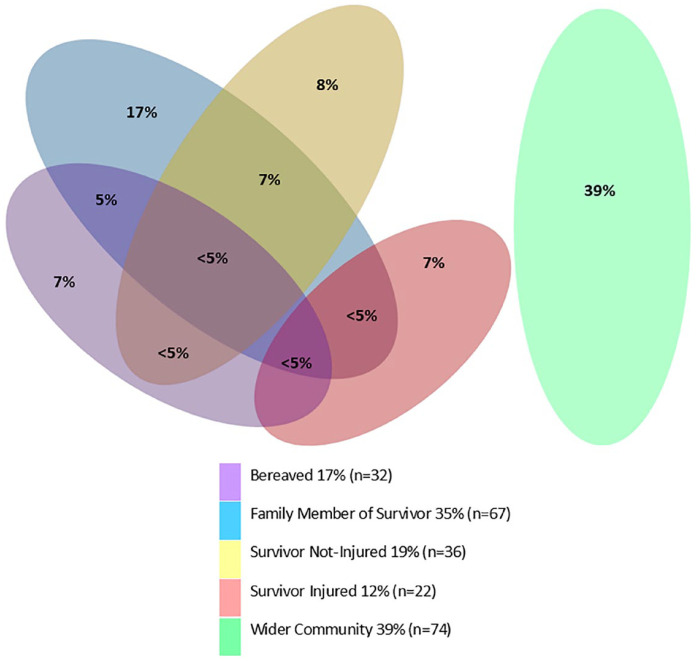
Exposure characteristics – percentage of sample in different exposure categories (*n* = 189). This Venn diagram is not drawn to scale. Exact percentages of <5% are suppressed to protect participant identity.

At the time of the attack, 31% of participants (39 male, 17 female) were present at or near one of the mosques, and 12% were physically injured. Many participants had family members who survived the attack (35%) or were killed (17%). A further 39% belonged to the wider Christchurch Muslim community. Some participants (23%) were in multiple exposure categories. The complex and interlinked participant and family member exposure characteristics are depicted in Figure 2.

### Mental health disorder profile

Based on the clinical interview, 47.6% of participants had at least one mental health disorder (anxiety disorder, major depressive disorder [MDD] or PTSD) at the time of interview, with 27% diagnosed with MDD, 24% with PTSD, and 28% with anxiety. In addition, 61% had experienced a mental health disorder at any time following the attack, with 43% meeting the criteria for MDD, 32% for PTSD, and 31% for anxiety disorder. Co-occurrence of these disorders was common, with 28% experiencing two disorders and 9% experiencing all three. Prior to the attack, 38% of participants had experienced at least one of the mental health disorders. Details of factors associated with mental health outcomes from the clinical assessment is reported in detail elsewhere (Bell et al., 2024).

### Self-reported psychological symptoms and functioning

Self-report measures showed results consistent with the clinical interviews (Table 3), with approximately 39% of participants reporting moderate/severe psychological distress (scoring ⩾ 25 on K-10), 39% indicating probable PTSD (>33 on the PCL-5), and 40% scored ⩽48 on the WHO-5. This is below the recommended cut-off of 50 for screening clinical depression, indicating that these people may be experiencing low levels of well-being and could be at risk for depression (Topp et al., 2015). Moderate or poor functioning was reported by 27% of participants on the WSAS, and 34% reported very high somatic symptoms (SSS-8). A substantial proportion (39%) of participants reported feelings of guilt or shame for surviving when others did not, and 8% reported being extremely bothered by these feelings.

**Table 3.** Self-reported psychological impact, well-being, and distress at assessment (*n* = 189).

	n	(%)
Psychological distress (K-10) (Kessler et al., 2003)		
Likely to be well (below 20)	88	(47)
Mild (20–24)	28	(15)
Moderate (25–29)	29	(15)
Severe (30 or above)	44	(23)
Well-being (WHO-5) (Topp et al., 2015)		
Otherwise	112	(59)
Poor well-being or potential depression (below 50)	77	(41)
Post-traumatic Stress Checklist (PCL-5) (Weathers et al., n.d.)^a^		
Otherwise	114	(61)
PTSD diagnosis suggested (from cut-off above 33)	74	(39)
Somatic symptoms (SSS-8) (Gierk et al., 2014)		
None to minimal severity (0–3)	28	(15)
Low (4–7)	32	(17)
Medium (8–11)	31	(16)
High (12–15)	34	(18)
Very high (16–32)	64	(34)
Work and social adjustment scale (WSAS) (Mundt et al., 2002)		
Sub-clinical (below 10)	101	(54)
Functional impairment (10–20)	36	(19)
Moderate or worse psychopathology (above 20)	50	(27)
Survivor guilt		
None	112	(61)
Bothered a little	32	(17)
Moderately bothered	14	(8)
Quite a lot	12	(6)
Extremely bothered	14	(8)

a Missing data for one participant.

### Quality of life, perceived discrimination and social connections

Table 4 describes findings relating to quality of life and social connections. Participants reported moderate levels of satisfaction (7.2/10) with their life as a whole, measured using Question one of the PWI (Lau et al., 2005). The lowest scores for the other questions of the PWI were observed in the domains of *future security* (6.6/10), *health* (6.7/10) and *feeling of safety* (6.7/10). Conversely, satisfaction with *religion* was notably higher, with a mean score of 8.5/10. The PWI was also modified to ascertain whether their satisfaction had increased, stayed the same, or decreased for each domain following the terrorist attack. A substantial proportion of respondents reported a decline in their satisfaction with their *life as a whole* (51%), *feelings of safety* (65%), *future security* (55%), and *health* (47%). In contrast, although many people indicated that their satisfaction levels remained constant, there was a noticeable increase in satisfaction with *religion* (49%). There were also modest increases with one-third reporting increased satisfaction with *feeling part of the community* (33%), and improved *personal relationships* (30%).

**Table 4.** Self-reported coping measures at assessment (*n* = 189), including changes in satisfaction with quality of life domains following the March 15 attacks, perceived discrimination and social networks.

		Increased	Stayed same	Decreased
	mean (SD)	*n* (%)^a^	*n* (%)^a^	*n* (%)^a^
Personal Well-being Index (PWI) (Lau et al., 2005)				
* Satisfaction with*:				
Life as a whole	7.2 (2.5)	34 (18)	57 (31)	95 (51)
Standard of living	7.4 (2.3)	32 (17)	113 (60)	43 (23)
Health	6.7 (2.5)	22 (12)	77 (41)	89 (47)
What you are achieving in life	7.0 (2.6)	37 (20)	94 (50)	56 (30)
Personal relationships	7.6 (2.5)	56 (30)	86 (45)	46 (25)
How safe you feel	6.7 (2.7)	24 (13)	41 (22)	124 (65)
Feeling part of the community	7.4 (2.3)	62 (33)	70 (37)	56 (30)
Future security	6.6 (2.6)	28 (15)	56 (30)	105 (55)
Religion or spirituality	8.5 (1.9)	92 (49)	74 (39)	22 (12)
Perceived Discrimination Scale (PDS) (Williams et al., 1997)			**n**	**(%)**
I was treated rudely or with disrespect			57	(30)
I received poorer service than others at a store or restaurant			29	(15)
People acted as though they were afraid or suspicious of me			45	(24)
I was treated unfairly			45	(24)
I was called names or insulted			34	(18)
I was threatened or harassed			25	(13)
I was excluded or ignored			42	(22)
Social Network Index (SNI) (Berkman and Syme, 1979)	Network size		n	(%)
0/1 (least social connections)	0–2 people		9	(5)
2	3–5 people		25	(13)
3	6–9 people		60	(32)
4 (most social connections)	10 or more people		95	(50)

a For each domain in the PWI, participants were asked to choose whether their satisfaction with that domain had increased, stayed the same or decreased after the March 15 attack. This has been reported as a percentage for each domain.

Half the participants (50%) reported experiences of perceived discrimination in the 3 months before the interview, often in multiple domains (RCOI, 2020; Sulaiman-Hill et al., 2021). This included being subjected to behaviours of fear and suspicion (24%), being treated unfairly (24%), excluded or ignored (22%), and encountering rudeness or disrespect (30%). However, community connectedness, measured by the SNI, showed that although 10% (*n* = 19) reported having no close friends, most people (74%) had between one and five people who they felt they could trust and confide in. As shown in Table 4, half of the sample reported a large social network (at least 10 people), which can likely be attributed to the social interactions from regular mosque attendance. This observation was reported by participants during interviews and supported by evidence documented in the scoring schedule.

### Post-traumatic growth and religious coping

Post-traumatic growth, measured by the PTGI, yielded a total mean score of 65.4 (out of 105) (see Supplementary File 1). The highest scores were observed in the domains *appreciation of life* (70%), *spiritual change* (69%), and *personal strength* (67%), with higher scores representing positive transformation as a result of March 15. Religious coping strategies were also frequently employed, with participants engaging in cognitive coping, behavioural coping and social coping techniques (see Supplementary File 1).

### Secondary stressors and support services

Participants reported multiple concerns, with financial issues (44%) and concerns about a family member’s psychological health (43%) being the most prevalent. Other concerns included children’s well-being (38%), family tensions (29%), employment issues (28%), housing problems (25%), immigration issues (15%) and court/legal processes (10%). Reports of multiple concerns were common, with almost half (46%) of the sample reporting three or more current concerns and 12% indicating six or more concerns.

At the time of the interview, 63% of participants reported having left Christchurch for a period following the terrorist attack, and 52% found that being away supported their recovery. Although the specific reasons for leaving Christchurch were not directly explored, anecdotal reports indicated that many participants chose to visit their countries of origin or to undertake religious pilgrimages in Saudi Arabia. Participants accessed a diverse range of activities and services, with general practitioners being the most frequently consulted service (76%), and they were considered helpful by 74% of the sample. Muslim social events were the most prevalent activity attended (71%) and were found to be helpful by 87% of those attending. See Supplementary File 2 for a summary of support services used by participants.

## Discussion

The Christchurch Muslim community, which was targeted in the March 15 terrorist attack, is characterised by its diversity and complex trauma exposures. The community comprises more than 40 different ethnicities, with varying social and linguistic backgrounds, migration pathways, and prior exposure to traumatic events. The sample in this study, which included representatives from 34 ethnicities, reflects the multicultural composition of this community. According to the 2018 Census (Statistics New Zealand, 2018), the Muslim population in Christchurch totalled 3942 individuals, with a gender distribution of 52% male and 48% female. Approximately 1000 were aged <15 years, and around 70% were aged 15–64 years. Despite the inherent challenges associated with such a diverse cohort, nearly 200 participants were successfully recruited for the study, with 89% of the sample consisting of individuals aged 18–60 years. This was largely achieved through early consultation, continuous engagement with the Muslim community and the involvement of Muslim RAs (Sulaiman-Hill et al., 2024).

Participants included those who lost family members, suffered injuries, witnessed the attack, had family members affected by these traumas, or were members of the Christchurch Muslim community. Emotional proximity to the attack was widespread with experiences such as the loss of close acquaintances, exposure to distressing imagery and repeated firsthand narratives from survivors adding complexity to the impact on the community. The nature of this incident and the heterogeneity of its victims mean that the physical and psychological exposure and sequelae are varied, complex, overlapping and potentially difficult to disentangle. Moreover, the aftermath extends beyond direct exposures to encompass secondary stressors (Stancombe et al., 2022), including financial concerns arising from injuries or loss of family members, immigration challenges to reunite families, and ongoing worries about repercussions on children and youth. This complex interplay of factors, including changing family dynamics, mental health concerns, and experiences of discrimination, collectively imposes a substantial psychological burden. This is evident in the elevated rates of mental health disorders and high scores on self-rating scales.

During the clinical interview, nearly half of the participants (48%) were diagnosed with current MDD (27%), anxiety disorder (28%), or PTSD (24%), and comorbidity was also common. This is consistent with previous research on mass shootings and terror attacks, which identified MDD and PTSD as common psychiatric sequelae (Lowe and Galea, 2017; Paz García-Vera et al., 2016). Questionnaire-based measures also revealed a substantial burden of morbidity for nearly half of the participants, with elevated symptom levels observed across several self-report measures. The psychological burden on individuals exposed to disasters, particularly those that are human-made or technologically created is known to be substantial (Neria et al., 2008), with victims of hate crimes and deliberate targeting being particularly vulnerable (Iganski and Lagou, 2016). The fact that this community was deliberately targeted due to their religion, makes this point particularly salient, especially as 50% of participants reported experiencing discrimination post-attack and in the 3 months preceding the interview. This high incidence of discriminatory experiences underscores the profound psychological and emotional toll on individuals who have already been subjected to targeted hostility, aligning with findings from other studies on Muslims in Western contexts (Dana et al., 2019; Viazminsky et al., 2022; Williams et al., 2022). While this heightened sensitivity to perceived incidents of discrimination may be attributed to the traumatic impact of the attack, it is also plausible that the social acceptability of racist remarks and behaviours diminished, at least temporarily, in the wake of the incident, potentially leading to fewer discriminatory situations than previously (Byrne et al., 2022). Research suggests that the temporal distance from terror events influences intergroup attitudes and perceptions (Choma et al., 2018), with societal attitudes eventually returning to pre-attack levels.

A second, more focused study, explored the impact of pre-existing factors on mental health outcomes in this sample (Bell et al., 2024). That analysis highlighted that prior exposure to traumatic events was associated with the subsequent development of PTSD, as well as an increased number of disorders post-attack. However, no significant association was found between prior exposure and the development of anxiety disorders or depression. Different exposures to the attack resulted in differential mental health sequelae. For example, direct exposure to the attack was associated with PTSD, while being bereaved was associated with PTSD, MDD and a greater number of disorders.

Participants faced numerous secondary stressors, including financial worries, family members’ mental health concerns, children’s well-being, employment and housing issues. These stressors compounded the psychological burden already present due to the attack, as observed elsewhere (Stancombe et al., 2022). These findings highlight the necessity for comprehensive psychosocial support services to address the diverse needs of the community (Stancombe et al., 2022).

Cultural perspectives and community resilience that were highlighted in the sample have been recognised as resources for supporting recovery in other collectivist cultures (Mao and Agyapong, 2021; Sippel et al., 2015). In addition, a strong religious belief and the use of positive faith-based coping strategies can help individuals make sense of traumatic incidents, and thereby enhance post-traumatic growth (Tedeschi et al., 2018; Wlodarczyk et al., 2016). Some researchers have reported that although physical proximity to a traumatic incident is predominantly associated with post-traumatic stress, emotional proximity may be linked to both post-traumatic stress and PTG (Wozniak et al., 2020). Deliberate introspection and rumination have also been recognised as contributors to PTG (Garcia et al., 2022) which aligns with our findings related to faith-based coping.

Participants who employ religious coping techniques, such as cognitive methods that integrate traumatic experiences within a broader spiritual framework, may find meaning and purpose in their suffering, by utilising their religious beliefs as a source of solace and comfort (Garcia et al., 2022; Wlodarczyk et al., 2016). For instance, framing the deceased as martyrs who are believed to be alive and occupying an elevated spiritual status in heaven is a prime example. Such contextualisation within a religious paradigm can promote coherence and purpose following adversity and is a key contributor to PTG (Taku et al., 2021).

The high mean total score for PTG among participants (65.4) compared to other studies (Steffens and Andrykowski, 2015) suggests substantial potential for personal development within the Christchurch Muslim community following the targeted attack. A systematic review, based on the PTGI measure (Steffens and Andrykowski, 2015), reported PTG scores ranging between 33.80 and 68.08 across various studies. The planned longitudinal component of this study aims to address gaps in the literature by examining the trajectory of PTG in this community, thereby providing valuable insights into their recovery and long-term outcomes.

This study also highlights the importance of social connections and interpersonal relationships for this predominantly migrant community. Based on comments during the interviews and anecdotal reports, participants sought support and advice from close friends, emphasising the significance of shared experiences and personal bonds in the aftermath of the attack. Quality-of-life measures also indicated the importance of personal relationships for participants well-being and attending Muslim community events was found to be beneficial for a large proportion of attendees. These findings corroborate previous research suggesting that support from individuals with similar experiences (Schildkraut et al., 2021), particularly within collectivist cultures (Mao and Agyapong, 2021; Tedeschi et al., 2018), can effectively validate victims’ experiences. Community-based networks, supported by personal relationships and faith-based coping methods, contribute to the search for meaning and acceptance. These findings align with the growing body of literature on PTG, which identifies communal coping and engagement in collective gatherings, particularly within religious communities (Tedeschi et al., 2018), as factors associated with positive personal development (Wlodarczyk et al., 2016).

### Strengths and limitations

The study has several important strengths that contribute to understanding the impact of a terrorist attack on a diverse community. By recruiting a wide range of participants, the research ensured the inclusion of various demographic and ethnic groups, thereby improving the generalisability of the findings. Furthermore, the trauma-informed methodology (SAMHSA, 2014) and the active involvement of the Muslim community in the leadership, design, development and conduct of the research process ensured cultural relevance and accurate representation of experiences. This not only fostered a sense of ownership and empowerment within the affected community but also promoted resilience and recovery.

The study design also addresses gaps in the existing literature, particularly the lack of evidence on safe and effective approaches to address mental health concerns following such events, especially for diverse groups (Genereux et al., 2019). In addition, the research responds to calls for longitudinal data (Lowe and Galea, 2017; Neria et al., 2008), long-term monitoring of psychological consequences (Genereux et al., 2019), and the integration of qualitative approaches (Schildkraut et al., 2021). A separate qualitative study has already been completed, and a follow-up study (the second measurement wave) will further evaluate the course and pattern of psychiatric disorders over time, but retention will be critical to maintain internal validity. The use of culturally appropriate, translated and validated measures, with cut-off points for self-report measures determined by comparison to standardised clinical interviews (Sulaiman-Hill et al., 2021) adds rigour and enhances the validity of the findings.

Despite these strengths, several limitations must be acknowledged. The modest sample size and convenience sampling method limit statistical power and generalisability. Selection bias could impact the external validity of the findings as not all the experiences of individuals affected by the attack may be captured in this study. In addition, potential biases in self-report measures, such as selection bias, social desirability bias, and the impact of emotional proximity, could influence participant responses, potentially exaggerating both negative and positive outcomes. It is also important to acknowledge the inherent limitations associated with self-report measures in terms of their sensitivity and specificity (Stuart et al., 2014). These limitations may contribute to an overestimation of the prevalence of clinical mental disorders (Scott et al., 2023), although the measures employed had good psychometric properties to mitigate some of these issues. In addition, the inclusion of diagnostic interviews offers a more accurate assessment of mental health disorders, providing a benchmark for the evaluation of screening instruments for use with similar populations.

## Conclusion

This study provides valuable insights into the impact of the Christchurch mosque attack on the affected community, highlighting the complexity of participant proximity, the substantial burden of morbidity experienced, and the importance of promoting social connections and positive outcomes to aid the recovery process for all those affected. The diversity of the population, their exposure to past trauma and varying exposures to this event underscores the need for nuanced, comprehensive psychosocial support services tailored to the unique needs of this community.

## Supplemental Material

sj-docx-1-anp-10.1177_00048674241276802 – Supplemental material for The psychosocial impacts of the 15 March terrorist attack on the Christchurch Muslim community: A descriptive, cross-sectional assessment Supplemental material, sj-docx-1-anp-10.1177_00048674241276802 for The psychosocial impacts of the 15 March terrorist attack on the Christchurch Muslim community: A descriptive, cross-sectional assessment by Ruqayya Sulaiman-Hill, Philip J Schluter, Sandila Tanveer, Joseph M Boden, Richard Porter, Ben Beaglehole, Shaystah Dean, Zimna Thaufeeg and Caroline Bell in Australian & New Zealand Journal of Psychiatry

## Notes

1. Referred to by the community as a Shaheed/Shuhada (pl) meaning martyr.

2. Royal Commission of Inquiry: A governmentally invoked body reserved for investigation into matters of the most serious public importance.
