# Supplementary material for: The psychosocial impacts of the 15 March terrorist attack on the Christchurch Muslim community: A descriptive, cross-sectional assessment
Source: Aust N Z J Psychiatry. 2024 Sep 8;58(11):977–89. doi: 10.1177/00048674241276802 (PMC11504151; doi:10.1177/00048674241276802)
Supplement: sj-docx-1-anp-10.1177_00048674241276802 – Supplemental material for The psychosocial impacts of the 15 March terrorist attack on the Christchurch Muslim community: A descriptive, cross-sectional assessment [file sj-docx-1-anp-10.1177_00048674241276802.docx]

**Supplementary File 1: Self-reported Post-traumatic Growth and Religious coping at assessment**

|  | **Min-Max range** | **Mean** | **SD** |
| --- | --- | --- | --- |
| *Post-traumatic Growth Inventory (PTGI)* ^a^ |  |  |  |
| *Mean score PTGI* | 0-105 | 65.4 | (23.0) |
| *Domain*: Relating to others | 0-35 | 21.2 | (8.1) |
| *Domain*: New possibilities | 0-25 | 13.7 | (6.3) |
| *Domain*: Personal strength | 0-20 | 13.3 | (5.3) |
| *Domain*: Spiritual change | 0-10 | 6.9 | (3.0) |
| *Domain*: Appreciation of life | 0-15 | 10.5 | (3.7) |
| *Religious Coping Scale (RCS)* ^b^ |  |  |  |
| *Mean score RCS* | 14-70 | 49.8 | (13.9) |
| *Domain*: Cognitive religious coping | 1-5 | 4.1 | (1.2) |
| *Domain*: Behavioural religious coping | 1-5 | 3.8 | (1.3) |
| *Domain*: Social religious coping | 1-5 | 2.6 | (1.1) |

**Note: ^a^missing data for 2 participants; ^b^missing data for 5 participants, 1 who was not religious**

**Supplementary File 2**: **Distribution of support services used and community activities engaged with, after the 15^th^ March attacks, by participants (n=189), and whether they were reported as being helpful in recovery for those who attended**

|  | | **Used/attended^g^** | | **Helpful^h^** | |
| --- | --- | --- | --- | --- | --- |
|  | | **n** | **(%)** | **n** | **(%)** |
| *Support services used* | |  |  |  |  |
|  | General practitioner and primary care | 144 | (76) | 106 | (74) |
|  | MSD case managers^a^ | 92 | (49) | 68 | (74) |
|  | Immigration advisors | 55 | (29) | 35 | (64) |
|  | Victim support workers | 86 | (46) | 67 | (78) |
|  | Māori agency support workers^b^ | 20 | (11) | 16 | (80) |
|  | Refugee & Migrant Resettlement Services | 29 | (15) | 18 | (62) |
|  | Court Victim Advisors | 53 | (28) | 41 | (77) |
|  | Police Family Liaison Officers | 68 | (36) | 58 | (85) |
|  | ACC^c^ | 39 | (21) | 26 | (67) |
|  | Specialist Physical health^d^ | 29 | (15) | 22 | (76) |
|  | Specialist Mental health^d^ | 20 | (11) | 13 | (65) |
|  | Counselling services | 46 | (24) | 31 | (67) |
|  | Other services | 8 | (4) | 5 | (63) |
| *Community activity* | |  |  |  |  |
|  | Islamic scholar events | 91 | (48) | 74 | (81) |
|  | Muslim social community events | 140 | (74) | 122 | (87) |
|  | Māori community events | 33 | (17) | 26 | (79) |
|  | Sports events or fitness programmes | 76 | (40) | 62 | (82) |
|  | Hydrotherapy | 17 | (9) | 9 | (53) |
|  | Pamper sessions | 31 | (16) | 25 | (81) |
|  | Parenting workshops/courses | 28 | (15) | 24 | (86) |
|  | Psychoeducation sessions | 17 | (9) | 11 | (65) |
|  | EMDR^e^ | 11 | (6) | 6 | (55) |
|  | Micronutrients^f^ | 8 | (4) | 6 | (75) |
|  | Life coaching sessions | 25 | (13) | 21 | (84) |
|  | Other | 27 | (14) | 21 | (78) |

**Note:^a^ MSD represents the Ministry of Social Development. This is a government organisation which provides social services and income support; A specialist multi-agency response team was established following the attacks to provide a single point of entry to support and access to government services. ^b^Kaupapa Māori providers (New Zealand Indigenous people lead); ^c^Accident Compensation Corporation which provides accident insurance cover for injuries to New Zealanders; ^d^Canterbury District Health Board was responsible for providing or funding the provision of most health services with the Canterbury region**; ^e^ **EMDR** **eye movement desensitisation and reprocessing sessions; ^f^ Some participants took part in a study looking at the potential benefits of micronutrient supplements on mental health; ^g^Percentage calculated from the total sample; ^h^Percentage calculated from those attending the service or activity.**
